# Mental health and support among young key populations: an ecological approach to understanding and intervention

**DOI:** 10.7448/IAS.18.2.19429

**Published:** 2015-02-26

**Authors:** Massy Mutumba, Gary W Harper

**Affiliations:** 1 Center for Sexuality and Health Disparities, Department of Health Behavior and Health Education, University of Michigan, Ann Arbor, MI, USA; 2 Joint Clinical Research Center, Kampala, Uganda; 3 Department of Health Behavior and Health Education, University of Michigan, Ann Arbor, MI, USA

**Keywords:** youth, adolescents, psychiatric disorders, psychosocial, HIV/AIDS, treatment, programmes

## Abstract

**Introduction:**

The patterning of the HIV epidemic within young key populations (YKPs) highlights disproportionate burden by mental disorders in these populations. The mental wellbeing of YKPs is closely associated with biological predispositions and psychosocial factors related to YKPs’ sexual and gender identities and socio-economic status. The purpose of this paper is to highlight sources of risk and resilience, as well as identify treatment and supports for mental health disorders (MHDs) among YKPs.

**Discussion:**

This paper utilizes Bronfenbrenner's Bioecological Systems Theory and the Social Stress Model to explore the risk and protective factors for MHDs across YKPs’ ecological systems, and identify current gaps in treatment and support for MHDs among these youth. We emphasize the fluidity and intersections across these categorizations which reinforce the vulnerability of these populations, the lack of concrete data to inform mental health interventions among YKPs, and the need to ground YKP interventions and programmes with human rights principles stipulated in the convention on the rights of a child.

**Conclusions:**

We put forth recommendations for future research and strategies to address the mental wellbeing of YKPs, including the need for integrated interventions that address the multiplicity of risk factors inherent in the multiple group membership, rather than single-focus interventions whilst addressing the unique needs or challenges of YKPs.

## Introduction

HIV infection disproportionately affects youth [1–3] , and the patterning of the HIV epidemic within young key populations (YKPs) underscores the role of mental health disorders (MHDs) in structuring vulnerability of these populations to HIV. For the purposes of this article, YKPs will be defined as sexual minority youth (including gay, bisexual, and lesbian youth and young men who have sex with men regardless of their sexual orientation identity); gender minority youth (specifically transgender and gender non-conforming youth); youth who inject drugs; youth involved in sex work; runaway and homeless youth; and detained or incarcerated youth.

MHDs may increase YKPs’ vulnerability to HIV, and/or alter the course of infection among those already living with HIV [4–6]. Among YKPs, MHDs have been linked to HIV risk behaviours such as early sexual debut, high numbers of sexual partners, low condom use, transactional sex, needle sharing, and drug/alcohol use [4–13]; lower uptake, adherence to, and retention in HIV care [14,15]; and increased risk of AIDS mortality [16]. Moreover, HIV infection also increases the risk of MHDs among YKPs [17,18].

## Epidemiology of MHDs among YKPs

Although adolescence and emerging adulthood is a time of relative positive physical health as measured by traditional indicators such as rates of mortality, chronic disease burden and hospitalizations, it is also a peak time for developing MHDs [19–21] and health-related challenges stemming from participation in high risk behaviours [22,23]. Studies have consistently reported higher rates of MHDs such as major depression, anxiety, conduct disorder, attention-deficit/hyperactivity disorder (ADHD), substance use disorder, alcohol dependence and abuse, suicide, and post-traumatic stress disorder (PTSD) among sexual minority youth [24–28], gender minority youth [25,27,29,30], youth who inject drugs [20,31], detained or incarcerated youth [32–36], runaway and homeless youth [37–39] and youth involved in sex work [40–42], relative to comparable youth populations. It is important to note that the higher rates of MHDs among YKPs are not due to any inherent dysfunction within these youth, but are closely associated with their membership in socially stigmatized minority groups that experience excessive stress in the form of prejudice-related stressful life events, discrimination, rejection and violence [43–45] .

Sexual minority adult populations have a two-fold excess in suicide attempts, and rates of depression, anxiety and substance use disorders are almost twice as high among sexual minorities compared to heterosexual populations [28]. Among sexual minority youth, a review of MHDs found that one third of participants met the criteria for any MHD including 17% for conduct disorder, 15% for major depression and 9% for PTSD [25]. Studies have reported even higher rates of MHDs among gender minority youth relative to comparable youth populations. A cross-sectional study of 515 gender minority persons found that 60% of participants were depressed; the prevalence of attempted suicide in this sample was 32% [29]. A cross-sectional study of 55 transgender youth found that 45% of participants had seriously considered suicide and 26% had attempted suicide [46], while another study of 571 male-to-female transgender persons in New York found that the lifetime prevalence of major depression among youth in this study was 54.7% [47].

More than two thirds of runaway and homeless youth meet the criteria for two or more MHDs including depression, conduct disorders, ADHD and PTSD [48–52]. Rates of attempted suicide among runaway and homeless youth who self-identify as sexual minorities range between 2 and 42% [26]. Detained or incarcerated youth are 10 times more likely to suffer from psychosis and depression compared to youth in the general population [32]. A nationwide review of 57 juvenile justice agencies (*N*=9, 819) found that 51.9% of youth met the criteria for a MHD; one third met the criteria for more than one disorder and about a quarter met the criteria for multiple clusters disorders. In this study, 20.4% reported anxiety, 27% reported disruptive behaviour disorder, 14% reported lifetime suicide attempts and 7.9% reported affective disorders [53]. Data on the mental health of youth involved in sex work are rare, but a study in Goa, India, found that 41.5% of female sex workers under 20 years of age had attempted suicide in the past three months [40]. Rates of physical and sexual violence among youth involved in sex work are high, ranging between 18 and 67% [54,55].

Current data point to sex/gender differences in prevalence of MHDs among YKPs. Gay/bisexual male youth have higher rates of panic and depression disorders, while lesbian/bisexual female youth have higher rates of substance abuse [56]. A study of sexual and gender minority youth found that transgender youth had a lower prevalence of all MHDs compared to gay/bisexual youth [25]. Among runaway and homeless youth, rates of drug abuse among were 10 times higher among male youth and 17 times higher among female youth as compared to youth in a nationally representative sample, and alcohol abuse was significantly higher among male youth [52]. Almost twice as many female runaway and homeless youth (25%) had attempted suicide at least once compared to male runaway and homeless youth (14%) [26]. Among detained or incarcerated youth, rates of major depression were twice as high among female youth compared to male youth (29% vs. 10.6%), while young men reported higher rates of psychotic illness (3.3% vs. 2.7%) [32]. These sex/gender differences underscore the diversity in experiences and needs within specific YKPs, which may have significant implications for intervention development. However, more studies are needed to elaborate on these differences.

### Cross-cutting issues

**Figure 1:**
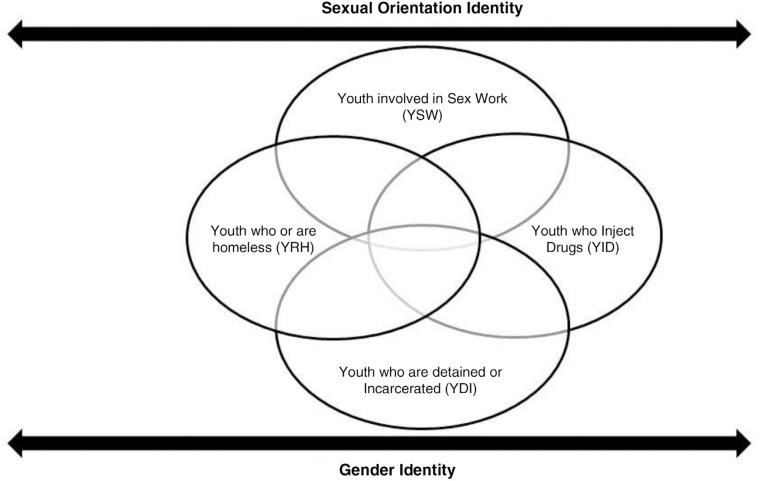
Intersection of group membership and identities among young key populations.

The needs and challenges of YKPs vary with their age, sex, race/ethnicity, gender identity, sexual identity, socio-economic status and geographic region. However, YKPs also share a host of socio-ecological experiences, broadly engendered by their sexual orientation and gender identities, which confer selective risks and vulnerabilities for MHDs and HIV. The categorizations of YKPs are not mutually exclusive (see Figure 1): there is high fluidity and intersections across these categories [57,58]. For example, approximately 30–45% of clients served in homeless youth services are sexual minority youth [59]; compared to heterosexual female youth, lesbian and bisexual youth are over-represented among detained or incarcerated youth [60,61]; homelessness is associated with greater risks for substance abuse [62]; and runaway and homeless youth who also self-identify as gay are more likely to report being tested or treated for HIV compared to bisexual or heterosexual youth [58,63] and are also more likely to engage in substance and alcohol abuse [64].

This intersectionality of oppressed identities and multiple memberships among YKP categories that experience social marginalization may increase the presence of MHDs. This is supported by the syndemic production theory, which posits that for some marginalized groups (e.g. sexual minority youth), there is a syndemic process of interacting physical and psychosocial challenges (e.g. HIV, substance use, depression, violence) that cause poor health outcomes within these populations [65,66]. For this reason, we emphasize the importance of developing interventions that address the intersectionality of social and cultural identities possessed by YKPs and the multiplicity of risk and resilience factors that may accompany membership in these various groups, rather than single-focus interventions.

## Discussion

### Theoretical framework

This paper utilizes two theoretical frameworks based on diathesis-stress models, to situate the epidemiology of MHDs among YKPs: Bronfenbrenner's Bioecological Systems Theory (BST) [67,68], and the Social Stress Model (SST) [69–71]. Generally, diathesis-stress models assert that all people have some level of pre-disposing risk factors (biological diathesis) for any given MHD, and that stress activates a diathesis, transforming the potential pre-disposition into an MHD [72–74]. BST is useful in understanding the linkages between biological factors and psychosocial factors in the development of MHDs among YKPs [69,70]. It proposes that an individual is continually impacted by four successive and interconnected levels of influence (i.e. microsystem, mesosystem, exosystem and macrosystem) over their life course; the biological diatheses and ecological stressors may act directly or synergistically to increase an individual's risk for MHDs. SST posits that one's disadvantaged position in the social hierarchy leads to more stressful conditions and fewer resources to counteract these stressors, resulting in greater rates of MHDs.

The *microsystem* comprises the complex relations between the developing person and the environments in the immediate settings containing the person. The *mesosystem* is a set of microsystems constituting an individual's developmental niche within a given period of development; mesosystems are more challenging to quantify and represent the assumption that microsystems do not function independently. The *exosystem* is composed of contexts that do not directly involve the developing person but have an influence on the person's behaviour and development. The *macrosystem* is the super ordinate ecological level of human development, involving culture, macro-institutions and public policy [75].

BST also provides a useful theoretical framework for understanding nested ecological system factors that influence the mental health and HIV risk/protective behaviours of YKPs, and the reciprocal relationships between youth and their environments. Both the BST and SST enable examination of youth within their social contexts, thereby allowing identification of contextually relevant cultural and developmental risk and protective factors.

**Figure 2:**
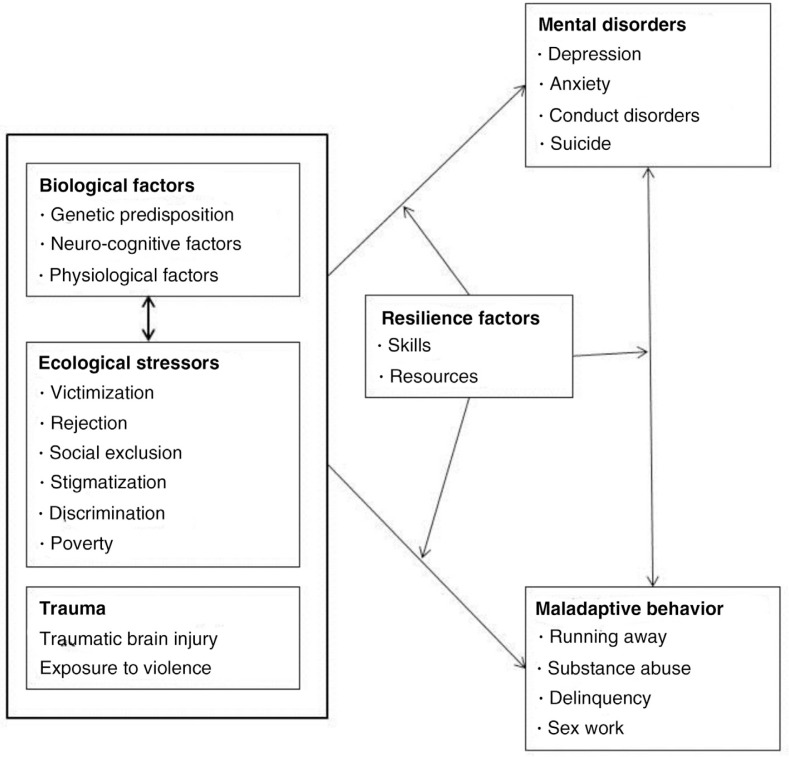
A framework using the Social Stress Theory to depict evolution of mental disorders among young key populations.

Figure 2 illustrates the adaptation of the SST to MHDs among YKPs. We argue that MHDs among YKPs result from their disadvantaged positions in the social hierarchy within their ecological systems, and this positioning is closely linked to their sexual orientation identity, gender identity and/or socio-economic status [76,77]. Consistent with our diathesis-stress framework, we argue that biological predispositions and ecological stressors may act independently, additively or synergistically to create MHDs and maladaptive HIV risk behaviours, and these pathways may be moderated by resilience factors [78]. This model highlights the reciprocal relationship between maladaptive behaviours and MHDs, which is also moderated by resilience factors.

### Risk factors

Microsystem factors could be sub-divided into intrapersonal and interpersonal factors. *Intrapersonal factors* include biological or cognitive factors that contribute toward certain abnormal states or conditions including genetic factors, inherited traits, neurological anomalies and patterns of psycho-physiological stress responses [73,74,79]. HIV may affect central nervous system structures involved in the regulation of emotion and behaviour, thereby increasing youth's risk of MHDs [80,81]. Additionally, normative developmental processes such as identity development and increased propensity for risk-taking, psychosocial distress manifested by self-esteem, poor self-image, hopelessness, helplessness and internalized homophobia may increase MHDs and HIV risk behaviours among YKPs [45,82–86]. Adolescence is a peak time for traumatic injuries, which, in turn, increase the risk of MHDs and HIV risk behaviours among youth [87,88]. MHDs such as depression, conduct disorder and PTSD and related dimensions of behaviour including coping strategies have been linked to diatheses such as genetic factors, depressogenic cognitive structures, traumatic brain injury and ecological stressors [72,73,87–89].

Interpersonal factors include experiences of victimization, family conflict, family/peer rejection, social isolation, poverty and housing instability [24,42,45,90–98]. Studies have found high rates of childhood maltreatment (physical and sexual abuse) among youth involved in sex work and runaway and homeless youth [99–102]. Childhood maltreatment, especially childhood sexual abuse, has been associated with alcohol use, delinquency and sexual risk behaviour [103–106] and MHDs such as depression and PTSD [107–109]. The pathways through which childhood physical and sexual abuse result in MHDs and HIV risk behaviours are not clearly elucidated but several authors hypothesize that these traumas could influence the development of maladaptive coping skills, maladaptive social information processing, and feelings of hopelessness, vulnerability and loneliness [110,111], leading to MHDs and HIV risk behaviours.

Exosystem factors include school and neighbourhood safety, neighbourhood poverty, stereotypes and representation of YKPs in communities, absence of caring adults, negative experiences with service providers, dearth of trained mental health providers, and geographical and financial barriers to accessing comprehensive and sensitive mental health services [85,112–116]. Exposure to violence, including war and civil strife, contributes significantly to MHDs, especially among youth in low-income countries [117,118]. The macrosystem factors include stigma, discrimination, social and economic marginalization, criminalizing or disenfranchising public policies, and cost of health care services.

All of these systems of influence act individually or synergistically to heighten YKPs’ risk for MHDs and HIV risk behaviours [42,45,112,119–122]. Different types of stressors (acute or chronic) may play different roles in the aetiology of MHDs and HIV risk behaviours. However, within the current research, there is a lack of theoretical attention to the nature and quality of the stressors, and the complex interactions through which biological diatheses and ecological factors influence the development of MHDs among YKPs.

### Resilience factors

Despite the host of powerful negative forces, not all YKPs have MHDs or engage in HIV risk behaviours. Many have multiple resilience factors – personal traits or characteristics of their social environment that protect young people from harm and reduce the likelihood of MHDs among vulnerable youth [123–125]. Intrapersonal resilience factors include high self-esteem, positive self-image, positive coping strategies, spirituality/religiosity, hopefulness, positive future expectations and participation in support or advocacy networks [85,90,98,123,126,127]. Interpersonal factors such as family and peers play an important role in youth development. Social support is generally hypothesized to be a protective factor that buffers individuals against the potential negative consequences of stressful events [128]. However, social support from family and peers may have differential effects for YKPs, including positive effects [129–133] or no effects at all [91,96,134]. For example, parental support may be more predictive of future MHDs than peer support [135,136]. Family connectedness and positive family acceptance have also been associated with positive mental health outcomes, particularly for sexual and gender minority youth who are grappling with issues of sexual orientation and gender identity [64,85,93,137–140]. Strong peer group affiliations may enhance risky health behaviours such as substance abuse and survival sex among runaway and homeless youth [141–143].

Exosystem factors include the availability of support from caring adults including teachers, case managers, programme facilitators and health providers; access to comprehensive youth-friendly social services with trained providers; non-discriminatory and anti-bullying policies in schools, homeless shelters and detention facilities; and child protection policies. These factors prevent MHDs, increase access to and utilization of health services, and promote YKPs’ ability to desist HIV risk behaviours [63,85,112,122,127,129,144]. Within the exosystem, organizations such as schools, churches, youth centres and health facilities are well positioned to provide safe environments and prevent MHDs among YKPs. For example, health providers could prepare youth and their families for changes related to pubertal development, understand and accept the gender and sexual identity of their children, provide parents/guardians with the skills needed to fully support YKPs and facilitate family re-integration (when appropriate). Schools, detentions centres, homeless shelters and foster homes can institute policies to prevent victimization of YKPs in these environments and advocate for the rights of YKPs. However, the success of these preventive actions requires providers who are knowledgeable and sensitive to the specific needs of YKPs.

### Programmes and interventions

Interventions to prevent or improve MHDs and HIV risk behaviours among YKPs are critical to addressing the HIV epidemic among youth. MHDs among youth are addressed through treatment with pharmacological agents or psychosocial interventions [145]. Current treatment guidelines discourage use of pharmacotherapy among children and adolescents [146]; rather pharmacological agents should only be prescribed if psychosocial interventions prove ineffective. However, compared to adults, the evidence base for management of MHDs and HIV risk behaviours among youth is less established. For example, depression and PTSD are some of the most common MHDs among YKPs [147], but evidence for the effectiveness of medications for treatment of these MHDs among adolescents remains elusive [146,148,149].

Globally, there is a paucity of programmes addressing MHDs and HIV risk among YKPs, and even fewer of these programmes exist in low- and middle-income countries [150]. Psychosocial interventions for management of MHDs among YKPs include interpersonal psychotherapy, cognitive behavioural therapy, behavioural therapy, psychodynamic therapy, structured physical activity programmes, relaxation training, problem-solving therapy and motivational interviewing. Within the adult literature, there is an extensive body of knowledge on the effectiveness of these psychosocial interventions [151] but even so, understanding the exact mechanisms by which these interventions achieve their effects and consensus over the relative effectiveness of different psychosocial therapies is lacking [145,152,153].

There is a paucity of interventions to prevent MHDs among YKPs. Universal and targeted prevention programmes have been developed to address alcohol and substance use and HIV risk behaviours among YKPs [154–156] , but results from systematic reviews of these interventions indicate that the majority do not obtain significantly better mental health outcomes compared to controls [150], and reductions in HIV risk behaviours, if realized, are often short-lived [150,154,157–160] . These findings suggest a need to re-consider strategies for engaging and promoting sustainability of behavioural gains among YKPs.

The persistent fragmentation of services, often with single-focus programmes targeting specific YKPs or MHDs, disregards the co-occurrence of MHDs among YKPs [161] and multiplicity of needs across these intersecting populations, thus limiting the efficacy of these interventions. Commonly cited components of integrated MHD services include comprehensive screening for all MHDs, development of a common treatment plan addressing all conditions, a multi-disciplinary team that includes a specialist in co-occurring disorders and psychosocial and pharmacological interventions, and services such as assertive outreach, coordinated care and supported employment [162].

The World Health Organization advocates for treatment of mental and psychosocial problems within primary care settings, but several concerns abound with this strategy including lack of organizational resources and expertise, gaps in provider knowledge regarding the developmental and mental health needs of YKPs, and lack of cultural competency in addressing needs of YKPs [163,164] . Additionally, YKPs may have significant challenges in accessing services within primary care settings, due to lack of health insurance coverage and concerns about confidentiality and privacy.

## Conclusions

Promoting the wellbeing of YKPs requires culturally and developmentally appropriate primary prevention interventions to eliminate or reduce risk factors for MHDs and HIV risk behaviours, and foster resilience factors throughout YKPs’ ecological environment. In addition, culturally and developmentally appropriate HIV care and mental health services are needed for youth living with HIV and/or MHDs, as well as secondary prevention interventions that promote healthy functioning and life course development for affected YKPs. All youth programmes and services need to address the intersectionality of marginalized identities and group membership often found among YKPs. They should be grounded in the latest theoretical and empirical data related to risk reduction and health promotion, and attend to the cultural and developmental needs of these youth.

While there is a growing body of knowledge regarding MHDs in some YKPs (e.g. sexual minority youth, runaway and homeless youth, detained or incarcerated youth), the literature on other populations such as gender minority youth and youth involved in sex work as well as YKPs in low-income countries continues to lag behind. The majority of studies on YKPs have been conducted in the United States; less is known about the psychosocial challenges or burden of MHDs among YKPs outside of the United States. This challenge is exacerbated by the lack of consistency in how MHDs are conceptualized and measured across countries and cultures [165–168], and differences in how adolescence is defined as a developmental period across settings [169,170]. Future research should focus on developing and validating mental health measures for non-US based populations and assessing the efficacy of these interventions in both US and non-US populations, keeping in mind the importance of tailoring interventions to local contexts.

Additional research is needed to increase understanding of key issues that influence the MHDs and HIV-related behaviours of YKPs, and to inform the development of effective interventions to address the unique needs of these young people. Of particular importance are studies elaborating on the complex pathways through which biological and ecological diatheses influence the development of MHDs and HIV risk behaviours among YKPs, the relative effects of the different types of stressors and appropriate strategies for management of MHDs in young people.

Interventions and research among YKPs would benefit from utilizing a BST framework for understanding the range of ecological factors that impact the MHDs and HIV-related risk and resilience of YKPs and in developing culturally and developmentally appropriate MHD and HIV-focused primary and secondary prevention interventions for YKPs. More research is needed to better understand the burden of MHDs in YKPs (especially outside of the US) and the interaction of MHDs and HIV-related risk and resilience. Such research should be sensitive to the multiple group membership of YKPs in often marginalized populations, addressing the multiplicity of risk and resilience factors across YKPs.

Given the high levels of stigma and discrimination experienced by YKPs globally, we argue that youth interventions should be rooted in the key human rights principles advanced in the convention on the rights of a child [171] including: (1) protection from physical and mental harm and exploitation; (2) utilization of evidence-based practices in establishing programmes and services for children; (3) provision of secure conditions that ensure dignity and promote self-reliance and (4) participation in decision-making processes taken in their regard. These human rights principles should supersede any social, cultural, political and other hegemonic ideologies, which may serve to oppress YKPs.

Below, we provide recommendations for practitioners and researchers on the best practices to promote the mental health and reduce HIV risk behaviours among YKPs:

1. *Consolidate youth services* to address the multiplicity of risk factors, and resulting MHDs and HIV risk behaviours. Develop partnerships across public, private and civil organizations to address the multiplicity of risk factors and special needs within YKPs, whilst attending to the individual needs of each youth. Such services should promote YKPs’ access to and sustained engagement in mental health services, HIV continuum of care and youth development programmes.
2. *Tailor programmes and interventions* to biological, cognitive, social and identity development stage of YKPs because there are wide variations in developmental differences between adolescents and young adults within YKPs.
3. *Expand training for providers in mental health*, particularly in low- and middle-income countries with a dearth of trained clinicians. This training should equip providers with the knowledge and skills to promote positive adolescent development and address the needs of YKPs through affirmative and respectful approaches.
4. *Create supportive environments* within programmes and services, and foster positive youth development by strengthening family, peers, school and community support systems. Family-centred interventions that enhance parent's/guardian's ability to connect with and support youth grappling with various psychosocial issues especially gender and sexual identity, and prevent risk factors such as family abuse, rejection and poor parent-youth communication and support are critical to preventing MHDs and HIV risk behaviours among YKPs.
5. *Develop youth capabilities* and *critical consciousness* by equipping youth with knowledge, skills and resources to counter their varied challenges [172] and provide opportunities for YKPs to participate in their socio-political environments.
6. *Develop and enforce formal child protection systems*, *policies and guidelines* in institutions such as schools and juvenile justice systems to prevent re-victimization of YKPs, and ensure access to mental health and HIV care services as well as positive youth development programmes within these contexts.
